# A Qualitative Study of a Pilot of Clinician Perspectives on the Delivery of Medicare Annual Wellness Visits for Patients with Dementia in an Academic Health Science Center in Texas

**DOI:** 10.1177/11786329261432894

**Published:** 2026-03-31

**Authors:** Huey-Ming Tzeng, Yong-Fang Kuo, Monique R. Pappadis, Elizabeth A. Hennessy, Maribel M. Marquez-Bhojani, Samuel V. David, Elise Passy, Mukaila A. Raji

**Affiliations:** 1 School of Nursing, The University of Texas Medical Branch (UTMB Health), Galveston, TX, USA; 2 Sealy Center on Aging, UTMB Health, Galveston, TX, USA; 3 Department of Internal Medicine-Geriatric Medicine Division, John Sealy School of Medicine, UTMB Health, Galveston, TX, USA; 4 Office of Biostatistics, UTMB Health, Galveston, TX, USA; 5 Department of Biostatistics and Data Science, School of Public and Population Health, UTMB Health, Galveston, TX, USA; 6 Department of Population Health and Health Disparities, School of Public and Population Health, UTMB Health, Galveston, TX, USA; 7 Alzheimer’s Association, Houston, TX, USA

**Keywords:** Medicare, aging, dementia, mild neurocognitive disorders, screening

## Abstract

**Background:**

Little is known about clinicians’ perspectives on the process and outcomes of Medicare Annual Wellness Visits (AWVs) in Medicare beneficiaries with mild cognitive impairment (MCI) or Alzheimer’s Disease and Related Dementias (ADRD).

**Objectives:**

We sought clinicians’ opinions on the impact of AWVs on health outcomes and disparity reduction for beneficiaries with MCI/ADRD.

**Design:**

Institute for Healthcare Improvement’s 4Ms framework of an age-friendly health system informed the design of this qualitative study of a pilot.

**Methods:**

We used convenience sampling and recruited clinicians from a single academic-health-science center’s catchment area in Texas, who billed for at least 1 AWV to participate in a one-time, one-on-one, semi-structured interview conducted via phone/Zoom. Participants verbally agreed to participate. This study met the federal regulations for a Quality Assessment project.

**Results:**

We interviewed 26 clinicians (17 female; 26 non-Hispanic, 12 White, 10 Asian, 4 Black; 16 in family medicine and 5 in internal medicine). Most agreed AWVs improve health outcomes (n = 23, 88.5%) and reduce health disparities for Medicare beneficiaries with MCI/ADRD (n = 20, 76.9%). The top three “what works” themes were: (1) non-primary care providers (eg, wellness nurses) streamline AWV delivery by screening patients, providing resources/support, and sharing abnormal findings with primary care providers (PCPs); (2) PCPs do AWVs themselves to be in alignment with issues identified; and (3) sufficient time allotted to learn what matters most to patients and caregivers. The top three “what does not work” themes were: (1) clinicians desire having a family caregiver present; (2) clinicians need the full hour for in-depth screenings and holistic care; and (3) clinics need on-site social workers to address nonmedical issues.

**Conclusions:**

Clinicians agreed that AWVs helped improve health outcomes and reduce health disparities. Tailoring AWV components by MCI/ADRD stage will optimize the visit, maximize health outcomes, and decrease disparities in access to care.

## Introduction

The Centers for Medicare and Medicaid Services (CMS) has offered free Annual Wellness Visits (AWVs) to older adults on Medicare (aged 65 years and older) since 2011.^1^ AWV components comprise physical and mental health risk assessment, medication review, personalized prevention plans, and advance care planning (ACP) consultation.^1-6^ AWVs allow primary care providers (PCPs) to spend focused time with Medicare beneficiaries, including patients with mild cognitive impairment (MCI) or Alzheimer’s Disease and Related Dementias (ADRD) (and their family caregivers when present), on identifying needs and providing support and resources for issues like reducing the risk of falls,^7,8^ engaging in ACP conversations,^9,10^ and preventing unplanned hospital admissions.^11^

In 2018, more than 9.5 million AWVs were performed.^1^ From 2018 to 2022, electronic health record data from 24 549 Medicare beneficiaries showed that 58.6% had 4 to 5 AWVs, 27.7% had 2 to 3 AWVs, and 13.7% had 0 to 1 AWVs.^12^ Beneficiaries were less likely to have had 4 to 5 AWVs if they were 85 years and older, Hispanic, or from socioeconomically disadvantaged areas.^12^ Another study found that males, adults of color and racial minority adults, and rural beneficiaries were less likely than others to use AWVs.^13^ AWVs enable the early detection of cognitive impairment and may potentially improve dementia care.^13^ Although AWVs could close gaps in preventive care, prioritize patient-provider relationship building, facilitate ACP, and improve primary care clinics’ financial incentives-related quality metrics, patients’ uptake of AWVs requires clinics to streamline their workflow (eg, sending a patient message explaining the benefits of AWVs via the patient portal).^14,15^ To maximize the impact of AWVs, healthcare delivery infrastructure and clinical protocols must be assessed to ensure convenient and equitable access to preventive services for people with MCI/ADRD.^16^ Soliciting PCPs’ perspectives on the delivery of AWVs for patients with MCI/ADRD is essential to maximize the impact of AWVs.

PCPs’ individual readiness to perform AWVs and their belief in the value of the AWV for early detection and planning are critical for performing AWVs consistently and with fidelity.^4,17^ PCPs were often concerned about the time pressure for addressing many of the elements in the AWV and patients’ needs related to their chronic or acute health issues.^14-18^ PCPs also expressed that they were motivated to adopt AWVs to meet patients’ needs and to receive financial incentives for their clinics.^14^ These clinicians valued AWVs as an opportunity to obtain a holistic view of patients outside the context of a problem-oriented visit, to align patients’ care with their wishes/values, and to influence patients’ behaviors. In addition, PCPs desired flexibility in AWV-related billing requirements to increase the adoption of AWV.^14^ Yet, some PCPs questioned the utility of AWVs, especially for patients with MCI/ADRD, which affected their engagement and follow-through.^14^

Previous studies^9,19,20^ have revealed that engaging patients with dementia and their families in primary care settings (eg, initiating ACP conversations, addressing hearing loss issues) often is more challenging compared with engaging those without dementia. More than half of the PCPs reported having little or no confidence in managing care for patients with MCI/ADRD (eg, having MCI/ADRD-related conversations with patients or their caregivers, interpreting cognitive assessment findings).^21,22^ PCPs with a geriatric focus or experience with patients with ADRD were more likely to fully implement AWVs and follow through with referrals and care planning (eg, screening for geriatric conditions, initiating ACP conversation).^6,19,23^ Clinicians also expressed challenges when providing care for Latino families caring for individuals living with dementia, because many Latinos had limited understating of dementia, often leading to a delay in seeking care, stigma associated with mental health issues, and preference for communicating with clinicians in Spanish, as well as the need to provide constant encouragement to Latino family caregivers to advocate for their family members with ADRD.^24^ Perales-Puchalt and associates^25^ interviewed PCPs who served Latino patients with ADRD to gain an understanding of the impact of the COVID-19 pandemic. They concluded that the experience of Latino families with ADRD resembled those of the general population.^25^

Little is known about clinicians’ perspectives on the required AWV components and efficiency of AWV delivery for Medicare beneficiaries with MCI/ADRD, and the impact of AWVs on their quality of care.^9,14,20^ Data derived from clinicians’ perspectives is essential to inform policymakers on potential AWV policy changes that can improve its delivery, especially for older patients living with MCI/ADRD—a fast-growing segment of the Medicare population.

### Purpose of the Study

This descriptive qualitative interview study sought to learn PCPs’ insights on AWVs for patients with MCI/ADRD at an academic health science center in Texas. We gathered the views of AWV providers (ie, physicians, nurse practitioners, and physician assistants, who have billed at least one AWV) on the process (ie, AWV components) and efficiency of AWV delivery, as well as whether AWV impacts the quality of care in patients with MCI/ADRD. We used the Institute for Healthcare Improvement’s (IHI) 4Ms framework of an age-friendly health system, which includes the four components of “what matters,” mentation, mobility, and medication, to guide study design.^26,27^ The main interview questions analyzed in this paper were: (1) agreement levels that AWVs can improve health outcomes and reduce health disparities in Medicare beneficiaries with MCI/ADRD; (2) what works and what does not work for older adults living with dementia who receive AWVs; and (3) what should happen to improve AWV delivery. This study used descriptive content analysis to generate themes. Regarding the importance of this study, we focused on patients with MCI/ADRD because data have shown that AWV affects the prevention of falls and fractures^8^ and the detection of changes in cognitive impairment (ie, early MCI diagnosis^13^). Understanding PCPs’ opinions on AWVs can guide health policy changes to improve AWV delivery and effectiveness.

This pilot clinician interview study used the clinician interview data collected in the parent project, which is funded by the National Institute on Aging, National Institutes of Health. In the parent project, we conducted two pilots, one solicited the perspectives of clinicians (the data used for the present pilot study), and the other solicited the perspectives of family caregivers, to understand “what works and what doesn’t” in AWVs for patients with MCI/ADRD in preparation for a larger national study. In this present study, we focused on PCPs’ viewpoints and aimed to develop and finalize major themes related to AWVs to inform a self-administered clinician survey in a larger national study involving up to 400 clinicians (part of the parent project).

### Conceptual Model: The Justification for Applying the 4Ms Framework

CMS announced that as of January 2025, Medicare’s Inpatient Prospective Payment Systems Hospital Inpatient Quality Reporting Program (HIQRP) will include a new age-friendly hospital structural measure that requires hospitals to report on whether they have relevant protocols in place.^28,29^ This new measure is based on the 4Ms framework. It includes five components of (1) eliciting each hospital inpatient’s healthcare goals, (2) medication management, (3) frailty screening, early detection, and intervention (ie, for cognitive impairment and delirium, mobility, and malnutrition), (4) recognizing and addressing social vulnerability, and (5) having age-friendly care leadership. This change is meant to reduce hospital readmission rates through improving the well-being of older adult patients post-hospitalization,^28-30^ such as healthy lifestyle changes for brain health.^31^ However, the CMS Hospital Inpatient Quality Reporting Program age-friendly hospital measure has limited focus on and attention to older adults with existing MCI/ADRD and does not include age-friendly measures for ambulatory care settings.

Access to high-quality geriatric care in primary care settings could be improved by incorporating the 4Ms framework into the AWV.^7,32^ Integration of this framework into the medical care of primary care clinics could lead to increased completion of AWVs, fall and depression screens, and advance care plans^33^ and could potentially benefit patients who suffer from cognitive impairment or multiple comorbidities.^34^ The most frequently reported barrier to implementing the 4Ms framework was the lack of clinicians’ buy-in.^35^ A unique aspect of this present study is framing the clinicians’ views through IHI’s 4Ms framework (“What Matters,” mentation, mobility, and medication) to study AWV use for community-dwelling Medicare beneficiaries with MCI/ADRD.^26,27,36^

**Figure 1.**
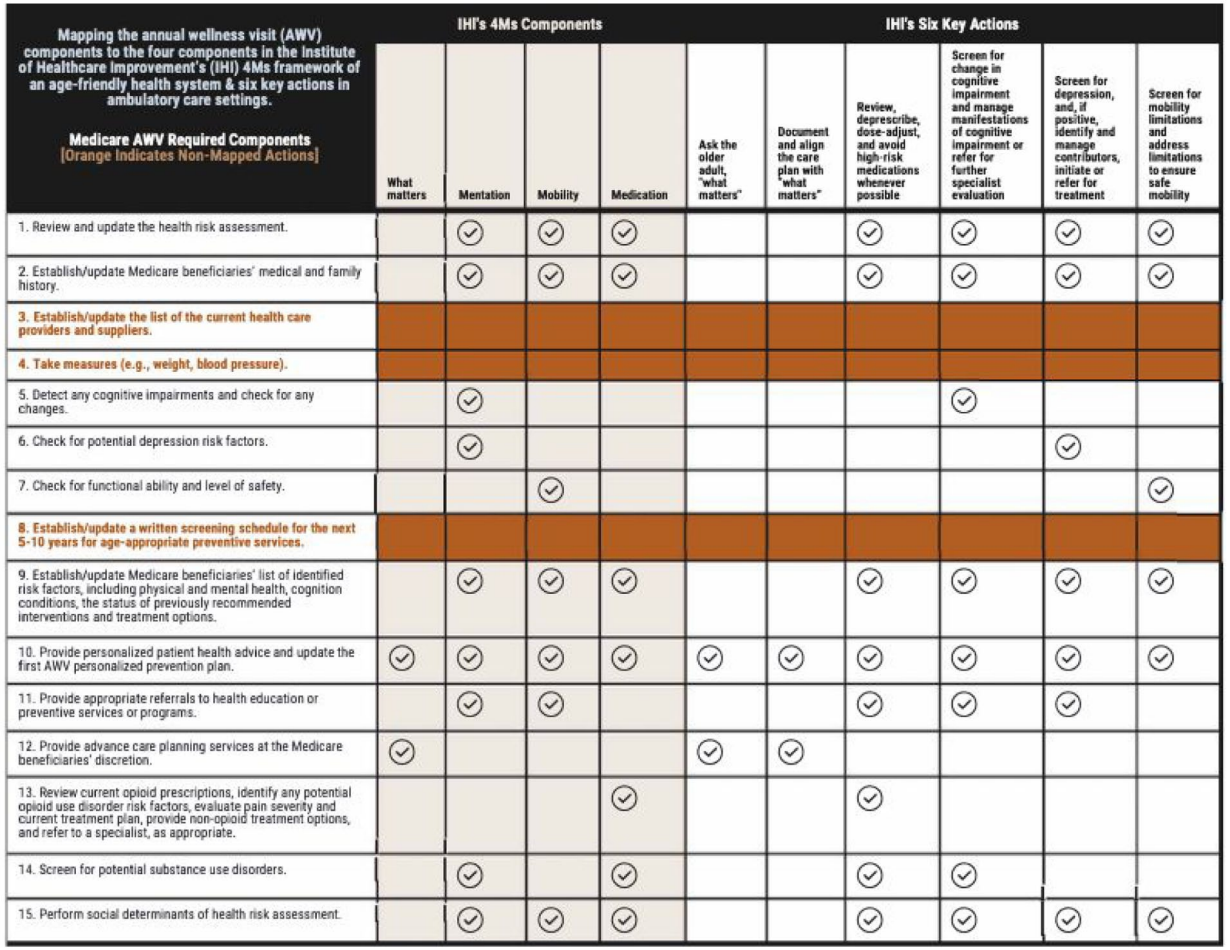
Mapping annual wellness visit (AWV) components to the four components in the Institute for Healthcare Improvement’s (IHI) 4Ms framework of an age-friendly health system and IHI’s six key actions in ambulatory care settings. Abbreviations: AWV, annual wellness visit; IHI, The Institute for Healthcare Improvement. IHI’s 4Ms components: The IHI’s 4Ms framework of an age-friendly health system includes the 4Ms components of “what matters,” mentation, mobility, and medication. IHI’s six key actions = IHI underscored that six key actions in ambulatory care settings should occur at least annually or after a change in condition. These actions are to: (1) ask the older adult “what matters”; (2) document and align the care plan with “what matters”; (3) review, de-prescribe, dose-adjust, and avoid high-risk medications whenever possible; (4) screen for change in cognitive impairment and manage manifestations of cognitive impairment or refer for further specialist evaluation; (5) screen for depression, and, if positive, identify and manage contributors, initiate or refer for treatment; and (6) screen for mobility limitations and address limitations to ensure safe mobility.

As shown in Figure 1, we mapped AWVs’ key components to IHI’s four 4Ms components and six key actions recommended by IHI for ambulatory care settings at least annually or after a change in condition.^26,27^ IHI’s six key actions include: (1) asking the older adult “What Matters”; (2) documenting and aligning the care plan with “What Matters”; (3) reviewing, de-prescribing, dose-adjusting, and avoiding high-risk medications whenever possible; (4) screening for change in cognitive impairment and managing manifestations of cognitive impairment or making referrals for further specialist evaluation; (5) screening for depression, and, if positive, identifying and managing contributors, initiating or referring for treatment; and (6) screening for mobility limitations and addressing limitations to ensure safe mobility.

We mapped 13 of the 15 AWV components^2^ to at least one of IHI’s 4Ms components and six key actions. We did not map three AWV components (#3, #4, and #8) to any of IHI’s 4Ms components or six key actions since those AWV components are intended for collecting essential information (eg, changes in health care providers, biological measures, validating whether patients have had the screenings for which they are eligible, and reminding patients about their screening schedule for the next 5 to 10 years for age-appropriate preventive services) to support any updates on the personalized prevention plan (#10). We also mapped two AWV components to “What Matters”: (#10) provided personalized patient health advice with an updated personalized prevention plan (ie, having patient/caregiver-clinician conversations to jointly identify or update feasible and practical health and wellness promotion recommendations or lifestyle changes that patients want and are able to engage), and (#12) discussion of ACP at the Medicare beneficiaries’ discretion (ie, clinicians solicit patients’ desire to develop or revise their ACP plans in follow-up ACP visits). CMS does not limit the frequency with which patients can revisit their ACP. Still, cost sharing applies outside the AWV.^2^ In short, AWV components cover at least one of the IHI’s four 4Ms components and six key actions, and “What Matters” is interwoven with the other three 4Ms components (mentation, mobility, and medication).

## Methods

### Design and Data Source

This descriptive, qualitative study was conducted using one-time, one-on-one, semi-structured phone or Zoom video conferencing interviews to gather opinions from clinicians who had billed at least one AWV to CMS. We sought to identify successful and unsuccessful elements of AWV delivery for Medicare beneficiaries with MCI/ADRD. This study employed descriptive content analysis to examine responses to open-ended questions. We followed the Consolidated Criteria for Reporting Qualitative Research (COREQ) guidelines.^37^

The project focused on investigating a specific health policy, Medicare Annual Wellness Visits, for people with dementia. The University of Texas Medical Branch’s Institutional Review Board (IRB) determined that this study met the federal regulations for a Quality Assessment/Quality Improvement project (the IRB did not provide the determination notification number). The classification of this study as a “Quality Assessment/Quality Improvement project” did not require IRB oversight; thus, we were expected to adhere to ethical research practices (eg, avoiding cold-call recruitment processes, ensuring protection of participants and reliability of data, and maintaining the confidentiality of participants in any publications). We conducted this study in accordance with the Declaration of Helsinki.

The protocol developed by the study team was carefully followed throughout the project. Any changes to the research processes that were previously discussed with the IRB were communicated to the IRB via email to ensure compliance with IRB regulations. All participants verbally agreed to participate in the interviews (written informed consent was not required because the one-time interview was conducted virtually via Zoom or phone) and to the interviews being recorded. An audio recording app (eg, available on iPads) and Zoom were used for both audio and visual recording to collect the data. The interviewer did not make field notes during and after the interview. Confidentiality of the participants was maintained, and only de-identified data were reported by the researchers for publication purposes. All research documents were accessible only to members of the study team.

As a quality assessment/quality improvement project, we followed the IRB’s rules and regulations related to participant recruitment. Clinicians interested in participating in the interview study were asked to take the initiative by contacting the study team via email, phone call, or mailing back the RSVP card (using a pre-paid business reply envelope provided by the study team).

### Sample

This pilot study aimed to conduct at least 25 interviews with clinicians and until data saturation was achieved (ie, when two consecutive interviews yielded no new codes in the codebook or themes, also known as inductive thematic saturation).^38^ The inclusion criteria were clinicians who had billed at least one AWV and were currently practicing as a family medicine physician, internal medicine primary care physician, geriatrician, nurse practitioner, or physician assistant. The exclusion criterion was that the clinician was not currently practicing. All clinicians who responded self-disclosed that they met the inclusion criteria and did not have the exclusion criteria.

As a pilot, we recruited participants from a sample of the catchment areas in Galveston County and adjacent counties in southeast Texas, from which patients of the University of Texas Medical Branch’s health system are drawn. This academic health center’s main campus is in Galveston County, Texas. In 2024, this academic health science center operated a total of 113 clinics, recorded 1 582 099 outpatient encounters, maintained 1031 hospital beds, and had 214 072 hospital patient days, resulting in 43 842 hospital discharges. As of September 2024, Galveston County had a total of 66 916 Medicare beneficiaries. Of these beneficiaries, 36 030 (53.8%) were female, and the racial distribution was 46 283 (69.2%) white, 8841 (13.2%) black, 8576 (12.8%) Hispanic, and 1727 (2.6%) Asian or Pacific Islander.^39^

To recruit participants, a total of 437 clinicians were identified using the publicly accessible CMS 2021 DAC national downloadable file and the 2018 to 2021 Texas Medicare Carrier File. We then randomly selected 250 potential participants from these 437 clinicians, regardless of their affiliation status with the University of Texas Medical Branch’s health system. The 250 sampled clinicians were contacted using mail (5 mailed attempts), phone calls (up to 5 attempts), and emails retrieved from public domains (up to 5 attempts). From this recruitment strategy, we recruited 7 participants over 5 months. Because of the low response, we next used convenience sampling to reach potential clinician participants through the University of Texas Medical Branch’s email platform (ie, daily announcements). In this way we recruited 19 participants over a period of 7 months.

Interviews took place from December 2023 to August 2024. Twenty-six clinicians responded to the recruitment materials and volunteered to participate through an online RSVP web form (n = 11, 42.3%), email (n = 7, 26.9%), regular mail (n = 5, 19.2%), or phone calls (n = 3, 11.5%). All agreed to schedule a one-on-one virtual interview via Zoom or phone and completed the interview. Participants gave verbal agreement to participate in the study before any interview questions were asked. Interviews lasted 15 to 40 minutes (average duration of 20 minutes) and were recorded. Only the interviewer and participant were present during the one-on-one virtual phone or Zoom interview. All interviews were completed in one sitting (no repeat interviews). Following the interview, participants received an emailed thank-you note; no monetary or other incentives were provided. Interview transcripts were not returned to participants for comments or correction, and participants did not provide feedback on the findings.

No relationship was established with the participants prior to the commencement of the study. Other than the recruitment materials, before the interviews participants did not know the interviewers (EAH, MMMB). The first author (HMT) trained both interviewers and provided coaching throughout the data collection period to ensure consistency across interviewers. The first author observed limited potential influence (eg, bias and assumptions) from the interviewers’ professional positions and training background on data collection and interpretation.

The final sample for this pilot study included 26 participants. As for the practice setting, 21 of them are current employees of the University of Texas Medical Branch’s health system, an academic health science center in Texas, 4 are affiliated with this academic health science center, and one has no affiliation with this center (Table 1).

**Table 1.** Brief Profiles of the Clinician Participants (n = 26).

ID	Sex	Ethnicity: Hispanic or Latino/a/x	Race	Credentials/specialties	Number of patients with MCI/ADRD	Age (years)	Years in practice	UTMB employee (Yes), affiliated with UTMB (Affi.), or not-affiliated with UTMB (No)	Geography/county of primary practice setting in Texas	Geography/primary practice area^
1	Male	Non-Hispanic	Asian	Internal medicine	1-25	49	21	Yes	Galveston	Metropolitan
2	Male	Non-Hispanic	Asian	Internal medicine—geriatric medicine	51-75	66	34	Yes	Galveston	Nonmetropolitan
3	Female	Non-Hispanic	White	Family medicine	26-50	65	17	Affi. (previous UTMB employee)	Harris	Metropolitan
4	Female	Non-Hispanic	Asian	Family medicine	26-50	67	17	Affi. (previous UTMB employee)	Fort Bend	Metropolitan
5	Female	Non-Hispanic	Black	Family medicine	26-50	40	7	Affi. (research collaborator)	Fort Bend	Metropolitan
6	Female	Non-Hispanic	White	Nurse practitioner—gerontology	More than 100	35	6	Yes	Galveston	Nonmetropolitan
7	Female	Non-Hispanic	White	Family medicine	26-50	43	10	Yes	Galveston	Nonmetropolitan
8	Female	Non-Hispanic	White	Family medicine	1-25	71	41	Affi. (scholarship donor)	Jefferson	Metropolitan
9	Male	Non-Hispanic	Asian	Family medicine	76-100	32	4	Yes	Galveston	Nonmetropolitan
10	Male	Non-Hispanic	White	Family medicine	1-25	35	10	Yes	Galveston	Nonmetropolitan
11	Female	Non-Hispanic	Asian	Family medicine	1-25	58	28	Yes	Harris	Metropolitan
12	Male	Non-Hispanic	Asian	Family medicine	1-25	50	18	Yes	Galveston	Metropolitan
13	Female	Non-Hispanic	White	Internal medicine—geriatric medicine, internal medicine	More than 100	60	34	Yes	Galveston	Metropolitan
14	Male	Non-Hispanic	White	Family medicine	More than 100	33	8	Yes	Galveston	Nonmetropolitan
15	Male	Non-Hispanic	Black	Family medicine, family medicine—adult medicine	1-25	42	12	Yes	Brazoria	Metropolitan
16	Female	Non-Hispanic	Black	Multi-specialty group, nurse practitioner—primary care, family medicine—geriatric medicine, internal medicine—hospice and palliative medicine	More than 100	52	11	No	Fort Bend	Metropolitan
17	Female	Non-Hispanic	Black	Internal medicine—geriatric medicine	26-50	52	4	Yes	Galveston	Nonmetropolitan
18	Male	Non-Hispanic	Asian	Family medicine	26-50	70	33	Yes	Galveston	Metropolitan
19	Female	Non-Hispanic	Asian	Family medicine	26-50	52	13	Yes	Harris	Metropolitan
20	Female	Non-Hispanic	Asian	Family medicine—geriatric medicine, family medicine	1-25	58	26	Yes	Galveston	Nonmetropolitan
21	Female	Non-Hispanic	White	Family medicine	51-75	78	50	Yes	Galveston	Nonmetropolitan
22	Female	Non-Hispanic	Asian	Internal medicine, preventive medicine—public health and general preventive medicine	26-50	37	7	Yes	Galveston	Metropolitan
23	Female	Non-Hispanic	White	Nurse practitioner—family	76-100	57	6	Yes	Galveston	Nonmetropolitan
24	Male	Non-Hispanic	White	Internal medicine	1-25	57	30	Yes	Galveston	Nonmetropolitan
25	Female	Non-Hispanic	White	Family medicine	26-50	59	24	Yes	Galveston	Nonmetropolitan
26	Female	Non-Hispanic	White	Internal medicine	1-25	47	17	Yes	Galveston	Nonmetropolitan

Abbreviations: ADRD, Alzheimer’s Disease and Related Dementias; MCI, mild cognitive impairment; UTMB, The University of Texas Medical Branch Health System.

^ Metropolitan versus nonmetropolitan practice location is based on the primary practice location’s zip code. Galveston County includes both metropolitan and nonmetropolitan areas.

### Measures

We developed a semi-structured interview guide that was based on the study’s purpose and IHI’s 4Ms framework. The interview guide included open-ended and multiple-choice questions using a Likert scale. The initial interview guide was pilot-tested with one geriatrician; we did not include this interview in the final analysis. We also sought community stakeholders’ insights on the questions in the guide from individuals on the AWV project’s designated community advisory board to help refine and finalize the interview guide.

The interview guide (Supplemental eFigure S1) included a framing statement, 10 main interview questions, and 8 demographic characteristics of participants (ie, credentials/specialties, total years of practice, estimated number of Medicare beneficiaries with MCI/ADRD in the past 12 months, metropolitan/nonmetropolitan practice location based on the primary practice location’s zip code,^40^ sex, ethnicity, race, and age).

The main interview questions (ie, the main and sub-questions) analyzed in this paper were: (1) agreement levels that AWVs can improve health outcomes and reduce health disparities in Medicare beneficiaries with MCI/ADRD (Q1); (2) what works and what does not work for older adults living with dementia who receive AWVs (Q7 and Q8); and (3) what should happen to improve AWV delivery (Q9).

We also analyzed dementia care-related questions: (1) priorities when it comes to the health of older adult patients with dementia (Q2); (2) challenges when it comes to the health of your older adult patients with MCI/ADRD (Q3); (3) what you do at the practice location that allows you to recognize and diagnose people in the early stage of dementia (Q4); (4) opinion on how dementia diagnosis could be streamlined and caught sooner in older adults (Q5); and (5) opinion of the new FDA-approved dementia care medications (Leqembi or Kisunla) (Q10). We included questions Q3 to Q5 because (a) there is an under-recognition of MCI in primary care, with 99% of clinicians missing MCI diagnoses,^41^ and (b) recent data show that AWV increases early MCI diagnosis, allowing those patients (and their care partners) the opportunity to be considered for use of the new FDA-approved anti-amyloid disease-modifying medications, which are approved for MCI due to AD or early AD.^13^

### Analyses

The interview included open-ended and multiple-choice questions using a Likert scale. The coded transcripts and responses to multiple-choice questions and demographic characteristic questions were entered into an SPSS^42^ file to generate descriptive statistics (eg, mean, standard deviation, frequency, and percent).

The responses to the open-ended questions were analyzed using a descriptive inductive content analysis method. The responses to the multiple-choice questions were entered into an SPSS^42^ data set. Exploratory analyses of the responses to the multiple-choice questions were descriptive (ie, frequencies). Due to the small sample size of 25, we did not pursue an analytical integration between the responses to open-ended questions and those to multiple-choice questions. The processes for conducting the descriptive inductive content analysis are as follows.

All interviews were recorded and transcribed manually by a third-party vendor in verbatim form. Two coders (HMT, a PhD-prepared registered nurse, and MMMB, a DNP-prepared registered nurse) independently conducted an initial descriptive inductive content analysis (using an inductive thematic coding process to develop the initial codebook) of the first three transcripts in Microsoft Word. These two coders discussed and agreed on the initial qualitative coding (the codebook that organizes the codes and includes short quotes from the transcripts) through consensus and applied the initial codes to the remaining transcripts. When analyzing the remaining transcripts, both coders verified new codes and checked for duplicates, with an agreement level of 93.6% across 297 codes. Two coders met via Zoom to compare their analyses and resolve any disagreements. To ensure consistency between the data presented and the findings, we included sample quotes from participants. Microsoft Word was used in the coding process and IBM SPSS^42^ for organizing the identified themes. We did not use qualitative software during the content analysis process.

After coding was complete, the two coders performed descriptive inductive content analysis on the identified codes to identify themes and thematic categories (ie, overarching themes) for the open-ended questions using Microsoft Word. Similar codes could be grouped into a single theme; one code could represent one theme. Thematic categories were developed based on the identified themes. The two coders met via Zoom to compare their analyses and resolve any disagreements on the themes and thematic categories. The identified themes were reviewed to ensure that each theme was clearly stated and self-explanatory. Two interpretation meetings were held to refine and agree upon the final included themes and thematic categories; the goal of the interpretation meeting was to weave together the identified themes and thematic categories into a coherent whole.

To connect the study findings with IHI’s 4Ms framework, we mapped the responses to Q2 (priorities when it comes to the health of your older adult patients with MCI/ADRD) with IHI’s four 4Ms components and six key actions, as well as with the main AWV components. For Q3, we discussed and finalized the themes, ensuring each theme was self-explanatory. In addition, we finalized the themes for Q4-Q5 and Q7-Q9, clarifying the meaning and shortening the statements to improve readability. For Q4-Q5 and Q7-Q10, there are fewer than 10 themes for each interview question, which are distinct from one another; therefore, we did not form thematic categories.

### Data Saturation

Data saturation was reached when two consecutive interviews did not yield any new codes or any new themes for the open-ended questions (ie, inductive thematic saturation).^38^ A total of 297 codes were identified from the 26 interview transcripts. For the last two transcripts, we identified 8 new codes (the 25th transcript: 1 from Q9 and 1 from Q10; the 26th transcript: 2 from Q3, 2 from Q4, 1 from Q5, and 1 from Q10). These new codes added details to the existing codes in the codebook, but they did not result in the emergence of new themes. As a result, we concluded that the data had reached saturation and ceased recruitment and data collection after the 26th interview.

## Results

### Demographic Characteristics

Tables 1 and 2 profile the 26 clinician participants: 17 (65.4%) were female and 26 (100%) were non-Hispanic adults; 12 (46.2%) were White adults, 10 (38.5%) were Asian adults, and 4 (15.4%) were Black adults. As for the specialties, sixteen clinicians (61.5%) practiced in family medicine, 2 (7.8%) in family medicine-geriatric medicine, 5 (19.2%) in internal medicine, and 3 (11.5%) in internal medicine-geriatric medicine. These clinicians had an average of 18.77 years of clinical practice experience (standard deviation = 12.47, range: 4-50). As for the geography, thirteen (50%) practiced in metropolitan areas and the other 13 practiced in nonmetropolitan regions. Nineteen (73.08%) had their primary practice settings in Galveston County, and 7 (26.9%) had their primary practice settings in an adjacent county. Most (n = 18, 69.2%) had seen <50 patients with MCI/ADRD in the past 12 months.

**Table 2.** Descriptive Information of Participant Characteristics (n = 26).

Characteristics	N	%	Mean (standard deviation), range
*Sex*			
Female	17	65.4	
Male	9	34.6	
*Age* (in years)			52.19 (12.92), 32-78
*Ethnicity*			
Hispanic or Latino/a/x	0	—	
Non-Hispanic or Non-Latino	26	100.0	
*Race* (choose all that apply)			
White	12	46.2	
Black	4	15.4	
Asian	10	38.5	
American Indian or Alaska Native	0	—	
More than one race	0	—	
Credentials/specialties* (choose all that apply)			
Family medicine	16	61.5	
Family medicine—adult medicine	1	3.8	
Family medicine—geriatric medicine	2	7.7	
Internal medicine	5	19.2	
Internal medicine—geriatric medicine	3	11.5	
Internal medicine—hospice and palliative medicine	1	3.8	
Multi-specialty group	1	3.8	
Nurse practitioner—family	1	3.8	
Nurse practitioner—gerontology	1	3.8	
Nurse practitioner—primary care	1	3.8	
Preventive medicine—public health and general preventive medicine	1	3.8	
*Total years of clinical practice experience*			18.77 (12.47), 4-50
<1 year	0	—	
⩾1 to <10 years	7	26.9	
⩾10 to <20 years	9	34.6	
⩾20 to <30 years	4	15.4	
⩾30 years	6	23.1	
*Primary practice area (converted from the zip code)* ^			
Metropolitan	13	50.0	
Nonmetropolitan	13	50.0	
*Number of patients with MCI/ADRD* (Question: In your best estimate, in the past 12 months, how many of your patients were older adults aged 65 and older with cognitive impairment?)			
1-25 patients	9	34.6	
26-50 patients	9	34.6	
51-75 patients	2	7.7	
76-100 patients	2	7.7	
More than 100	4	15.4	

Abbreviations: ADRD, Alzheimer’s Disease and Related Dementias; MCI, mild cognitive impairment.

* Centers for Medicare and Medicaid Services. *Medicare Physician and Other Practitioner Look-up Tool*. Available from: https://data.cms.gov/tools/medicare-physician-other-practitioner-look-up-tool; https://npiregistry.cms.hhs.gov/search.

^ Metropolitan versus nonmetropolitan practice location is based on the primary practice location’s zip code.

### Clinician’s Perspectives on the Role of the AWV in Improving Health Outcomes and Reducing Health Disparities

Table 3 (with selected quotes) shows that most participants agreed that AWVs improved health outcomes (n = 23, 88.5%) (Q1.a). Most participants (n = 20, 76.9%) also agreed that AWVs reduced health disparities for Medicare beneficiaries with MCI/ADRD (Q1.b). We also observed that all 20 participants who agreed that AWVs reduced health disparities also agreed that AWVs improved health outcomes. Among the other 6 participants (23.1%) who did not agree that AWVs reduced health disparities, three agreed that AWVs improved health outcomes.

**Table 3.** Summary of Analysis of the Clinician Interviews: Clinicians’ Opinions of Annual Wellness Visits (AWVs) for Patients with MCI/ADRD (Question #1, Multiple-Choice Sub-Questions) (n = 26).

Questions (multiple-choice questions)	The response options	Frequency (%)	Mean (SD)
a. Based on your best estimate, do you agree that AWVs can improve the health outcomes of your older adult patients with dementia?	1 = Strongly disagree 2 = Disagree 3 = Nether agree/disagree 4 = Agree 5 = Strongly agree	2 (7.7) 0 (—) 1 (3.8) 12 (46.2) 11 (42.3)	4.15 (1.08)
*Quotes from participants’ comments related to their responses (3 themes)*: – Providing a venue to facilitate a conversation with shared decision-making on medical and nonmedical issues like healthy lifestyle for the patient with MCI/ADRD (TC#1) *“Patients with dementia are at risk for a lot of things, physical, emotional, mental health. When we [clinicians] do annual wellness, we capture issues based on the questionnaire. We have a conversation with shared decision-making on how their healthcare delivery can best benefit the patient and their loved ones to maintain and sustain a healthy lifestyle and living.” [#15, responded “strongly agree”] “During AWVs, we discussed the patient’s medications and what else could be done to make the patient a little more independent. Some of the patients live by themselves. We can discuss these non-medical issues during AWVs.” [#2, responded “strongly agree”]* – Providing a venue to facilitate a conversation with about advanced care planning for the patient with MCI/ADRD (TC#1) *“I do find AWV beneficial, for example, having conversations about advanced care planning and advanced directives.” [#22, responded “neither agree/disagree”]* – Benefiting patients with mild dementia, but not patients with severe dementia (TC#4, TC#5, TC#6) *“If the patient has mild dementia, I would strongly agree [that AWVs improve health outcomes]. But if the patient has more severe dementia, I would say most of the time it’s not [strongly disagree]. Family caregivers don’t want to do any proactive things. They [family caregivers] just want to manage symptoms because those patients, if they are overall in good health, may still have like 8, 10 years to go. We do a lot of screening for nutrition and depression. If they [patients] are malnourished or have depressed mood on top of dementia, they will have more problems. AWVs will not prolong their [patient’s] life, but I [clinician] would rather have them [patients] walking than be debilitated in bed.” [#13, responded “strongly agree”]*	(TC#1)		
b. Based on your best estimate, do you agree that AWVs can reduce the health disparities (eg, access to care) of your older adult patients 65 years and older and with dementia?	1 = Strongly disagree 2 = Disagree 3 = Nether agree/disagree 4 = Agree 5 = Strongly agree	2 (7.7) 0 (—) 4 (15.4) 13 (50.0) 7 (26.9)	3.88 (1.07)
*Quotes from participants’ comments related to their responses (2 themes)*: – Helping with identifying needs and alert clinicians to refer the patients and their family caregivers to the social worker for assistance (TC#6) *“From the wellness nurse’s visit report, I can pick up on more social issues and help in that regard [e.g., transportation, food, and financial difficulties]. We have patients that we list as noncompliant because they’re always missing appointments. We don’t know why. But when I look at the wellness visit notes, it’s all there, which helps me redirect the patients and their family caregivers to the social worker or see what resources I have to provide.” [#17, responded “agree”]* *“AWV does help me identify health disparities (e.g., housing insecurities), but we do not always have resources to address those health disparities. Sometimes we identify problems, and then we cannot fix them.” [#25, responded “agree”]* – Helping with access to care to decrease health disparities (TC#1, TC#6) *“AWV is a way to interact with patients and communicate with patients who never seek healthcare.” [#11, responded “strongly agree”]*			

Abbreviations: ADRD, Alzheimer’s Disease and Related Dementias; AWV, annual wellness visit; MCI, mild cognitive impairment; TC, thematic category.

Across Tables 3 to 5 (questions #1a, 1b, 3, 7-9), we identified 7 thematic categories (abbreviated as TC), as the overall thematic structure, which are: (TC#1) providing additional time to attend to patients’ issues and needed support; (TC#2) engaging primary care providers (PCPs) and trained non-PCP providers in expanding the AWV delivery capacity; (TC#3) scheduling and increasing patient/caregiver uptake needing PCPs’ in-person explanations of AWVs and encouragement during maintenance visits; (TC#4) assisting patients/caregivers in completing the AWV assessment questionnaire before AWVs to allow time for AWV providers to identify needs and develop/update the personalized prevention plan within the allotted time for each AWV; (TC#5) experiencing challenges in assessing patients’ risk factors and developing/updating the personalized prevention plan without a trusted caretaker to accompany patients with MCI/ADRD; (TC#6) assessing patients’ social and support needs and providing timely access; and (TC#7) affiliating with an existing referral network or health system providing timely access to clinical or non-clinical referrals or services. We included the corresponding TC number (TC#) after each theme.

### What Works and Does Not Work? What Should Happen to Improve AWVs?

Table 4 summarizes clinicians’ viewpoints about AWVs for Medicare beneficiaries with MCI/ADRD (Q7-Q9). The top three themes for what works (Q7) were: (1) non-PCP providers (eg, wellness nurses) streamline AWV delivery by screening patients, providing resources/support, and sharing abnormal findings with PCPs (n = 5, 19%); (2) PCPs perform AWVs themselves so they are in alignment with the issues identified by patients (n = 4, 15%); and (3) sufficient time is allotted to learn patients’ care goals related to what matters most to them and their family caregivers (n = 4, 15%).

**Table 4.** Summary of Analysis of the Clinician Interviews: Clinicians’ Viewpoints on the Annual Wellness Services for People with Dementia (n = 26; Open-Ended Questions #7-9).

Themes (abstracted from the open question)	Themes found in the transcripts (yes/no)	Frequency of each theme found in the transcripts (%)
*Please share with me your opinion on what works in your practice or health care system for older adults living with dementia who receive Annual Wellness Visits (Question #7; 5 themes)*		
1. Non-PCPs (eg, registered nurses) streamline AWV delivery by screening patients, providing resources/support, and sharing abnormal findings with PCPs (TC#2)	Yes	5 (19)
	No	21 (81)
2. PCPs do AWVs themselves to be in tune with the issues identified (TC#1, TC#2)	Yes	4 (15)
	No	22 (85)
3. Sufficient time allotted to learn patients’ care goals: what matters most to them and their family caregivers (risk reduction, medications, wellness, complete assessments) (TC#1)	Yes	4 (15)
	No	22 (85)
4. Health system affiliation provides timely access to clinical and other services for patients with dementia (TC#7)	Yes	3 (12)
	No	23 (88)
5. PCPs explain the importance of AWVs and encourage patients to schedule (TC#3)	Yes	2 (8)
	No	24 (92)
*Please share with me your opinion on what does not work in your practice or health care system for older adults living with dementia who receive Annual Wellness Visits (Question #8; 7 themes)*		
1. Clinicians desire having a family caregiver present (ie, a reliable source for the patient’s medical history, including vaccinations) (TC#5)	Yes	4 (15)
	No	22 (85)
2. Clinicians need the full hour to conduct in-depth screenings and provide holistic care (TC#1)	Yes	4 (15)
	No	22 (85)
3. Clinics need on-site social workers to address nonmedical issues (eg, transportation, food insecurity, care coordination, call/checkup on patients, having a living will) (TC#6)	Yes	4 (15)
	No	22 (85)
4. Generic notifications to attend a Medicare AWV are ineffective. Patients need PCPs’ push (TC#3)	Yes	2 (8)
	No	24 (92)
5. Patients and their family caregivers need help understanding the copay and types of services provided (TC#3)	Yes	2 (8)
	No	24 (92)
6. Clinicians can identify needed resources but cannot help patients/family caregivers obtain them (TC#6)	Yes	2 (8)
	No	24 (92)
7. Patients with dementia need more than annual monitoring, such as a monthly phone call from nursing staff (TC#1)	Yes	2 (8)
	No	24 (92)
*Please share with me your opinion on what should happen to improve Annual Wellness Visit delivery for older adults living with dementia (Question #9; 9 themes)*		
1. Clinics should require a trusted caretaker to come to AWVs with dementia patients to advocate, answer questions, and provide medical history (TC#5)	Yes	6 (23)
	No	20 (77)
2. Trained non-PCP providers (eg, RNs and non-prescribers) could do more AWVs (TC#2)	Yes	4 (15)
	No	22 (85)
3. Clinic staff should call patients/family caregivers to tell them what to expect from AWVs and what to bring with them (eg, medications) (TC#3, TC#4)	Yes	3 (12)
	No	23 (88)
4. Clinics should help patients/family caregivers complete the questionnaire if needed (TC#4)	Yes	3 (12)
	No	23 (88)
5. Clinics could be offered AWVs via telehealth or at other sites (eg, patient’s home, assisted living, or memory care facility) (TC#1, TC#2)	Yes	3 (12)
	No	23 (88)
6. Medicare should increase the minimum time for AWVs to allow clinicians to finish the protocol without rushing (TC#1)	Yes	2 (8)
	No	24 (92)
7. Reimbursement should allow for additional services (eg, acute medical issues requiring timely treatment, such as high blood pressure) (TC#1)		
	Yes	2 (8)
	No	24 (92)
8. Clinics need increased social work support to help patients/family caregivers navigate the health system and community resources (eg, financial support for copay) (TC#6)	Yes	2 (8)
	No	24 (92)
9. Clinics should call family caregivers (eg, 6 weeks after the AWV) to follow up on recommendations (TC#1)	Yes	2 (8)
	No	24 (92)

Abbreviations: ADRD, Alzheimer’s Disease and Related Dementias; AWV, annual wellness visit; MCI, mild cognitive impairment; PCP, primary care provider; TC, thematic category.

Across Tables 3 to 5 (questions #1a, 1b, 3, 7-9), we identified 7 thematic categories (abbreviated as TC), as the overall thematic structure, which are: (TC#1) providing additional time to attend to patients’ issues and needed support; (TC#2) engaging primary care providers (PCPs) and trained non-PCP providers in expanding the AWV delivery capacity; (TC#3) scheduling and increasing patient/caregiver uptake needing PCPs’ in-person explanations of AWVs and encouragement during maintenance visits; (TC#4) assisting patients/caregivers in completing the AWV assessment questionnaire before AWVs to allow time for AWV providers to identify needs and develop/update the personalized prevention plan within the allotted time for each AWV; (TC#5) experiencing challenges in assessing patients’ risk factors and developing/updating the personalized prevention plan without a trusted caretaker to accompany patients with MCI/ADRD; (TC#6) assessing patients’ social and support needs and providing timely access; and (TC#7) affiliating with an existing referral network or health system providing timely access to clinical or non-clinical referrals or services. We included the corresponding TC number (TC#) after each theme.

The top three themes of what does not work (Q8) were: (1) clinicians desire having a family caregiver present (n = 4, 15%); (2) clinicians experience time pressures and need a full hour to conduct in-depth screenings and provide holistic care (n = 4, 15%); and (3) clinics need on-site social workers to address nonmedical issues (n = 4, 15%) (Table 4).

The top five themes on what should happen to improve AWVs for Medicare beneficiaries with MCI/ADRD (Q9) were: (1) clinics should require a trusted family caregiver to come to AWVs with patients with dementia to advocate, answer questions, and provide their medical history (n = 6, 23%); (2) trained non-PCP providers (eg, RNs and non-prescribers) could do more AWVs (n = 4, 15%); (3) clinic staff should call patients/family caregivers in advance to tell them what to expect from AWVs and what to bring with them to the appointment (eg, medications) (n = 3, 12%); (4) clinic staff should be available to assist patients/family caregivers to complete the questionnaire if they need help (n = 3, 12%); and (5) AWVs could be offered via telehealth or at other sites (eg, patient’s home, assisted living, or a memory care facility) (n = 3, 12%) (Table 4).

### Health Priorities for Patients with MCI/ADRD

**Figure 2.**
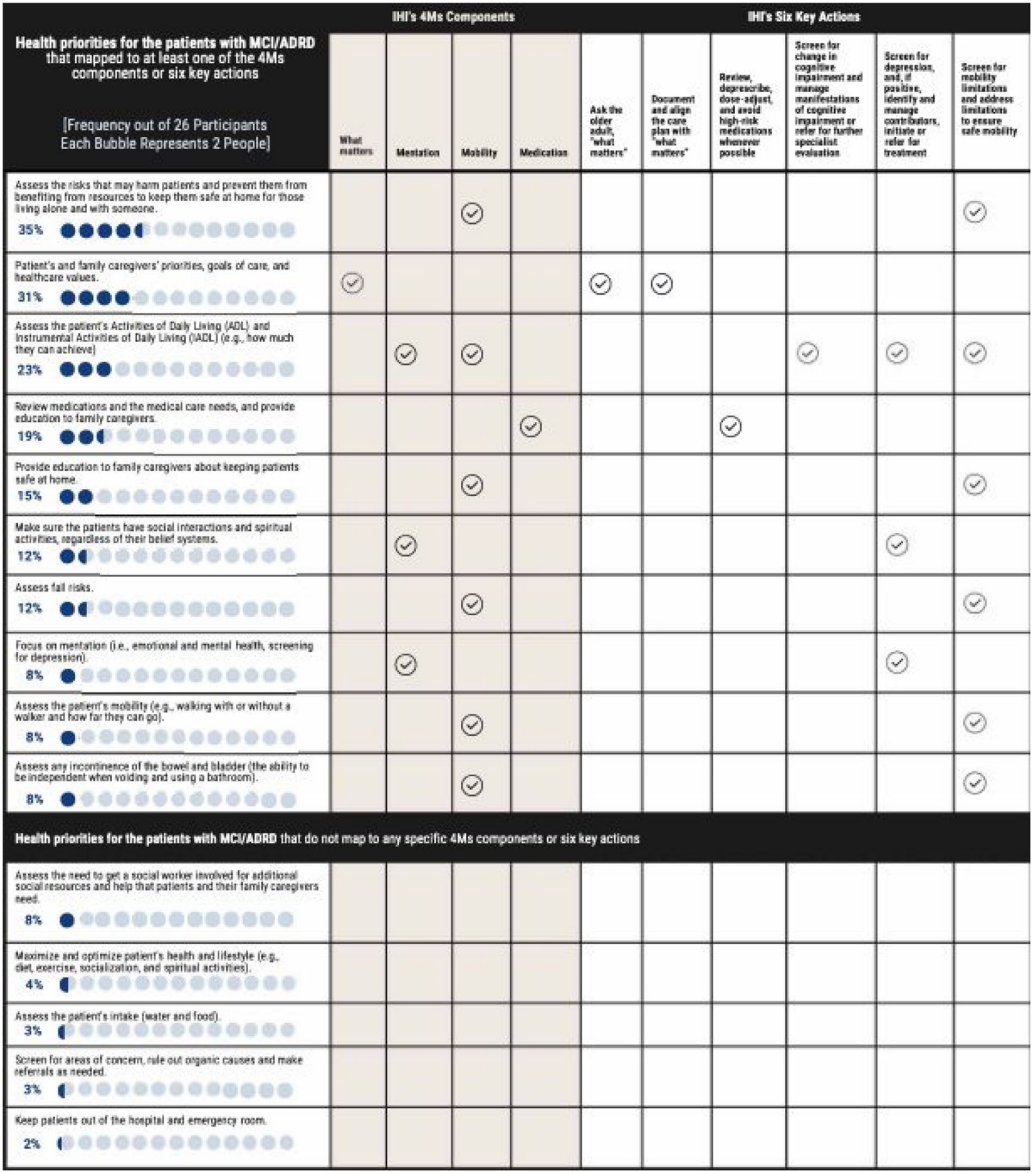
Mapping clinicians’ priorities for patients with MCI/ADRD (Question #2, one open-ended question about priorities when it comes to the health of older adult patients with dementia) to the components in IHI’s 4Ms framework of an age-friendly health system and IHI’s key actions in ambulatory care settings. Abbreviations: ADRD, Alzheimer’s Disease and Related Dementias; IHI, The Institute for Healthcare Improvement; MCI, mild cognitive impairment. 4Ms components: The IHI’s 4Ms framework of an age-friendly health system includes the 4Ms components of “what matters,” mentation, mobility, and medication. IHI’s six key actions = IHI underscored that six key actions in ambulatory care settings should occur at least annually or after a change in condition. These actions are to: (1) ask the older adult “what matters”; (2) document and align the care plan with “what matters”; (3) review, de-prescribe, dose-adjust, and avoid high-risk medications whenever possible; (4) screen for change in cognitive impairment and manage manifestations of cognitive impairment or refer for further specialist evaluation; (5) screen for depression, and, if positive, identify and manage contributors, initiate or refer for treatment; and (6) screen for mobility limitations and address limitations to ensure safe mobility.

As shown in Figure 2, 15 health priorities were identified in Q2 (priorities when it comes to the health of older adult patients with dementia). We mapped these priorities to IHI’s 4Ms components and 6 key actions; 10 of the health priority themes were mapped to at least one of IHI’s 4Ms components and 6 key actions.^26,27^ We did not map five of the identified themes (listed in the lower part of Figure 2) to any of the IHI’s 4Ms components and 6 key actions because these five priorities appeared to be relatively general and related to overall health issues and were intertwined with all the framework’s components and actions.

**Figure 3.**
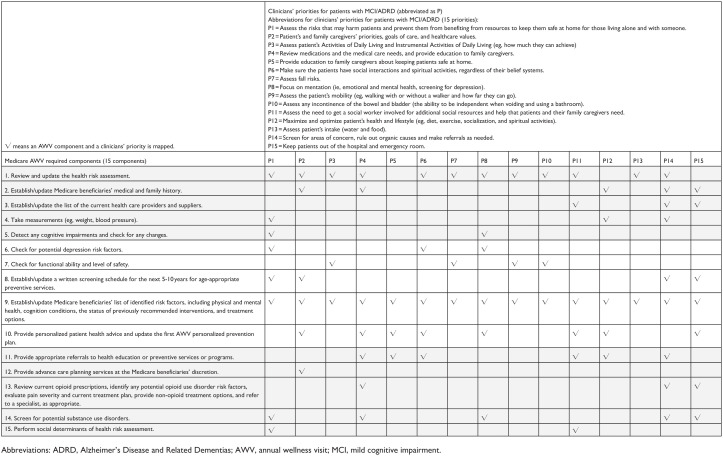
Mapping clinicians’ priorities for patients with MCI/ADRD to the AWV components (Question #2, one open-ended question about priorities when it comes to the health of older adult patients with dementia) to the AWV components.

We also mapped clinicians’ priorities for patients with MCI/ADRD to the AWV components (Figure 3). All 15 identified priorities were mapped to at least one AWV component. Observing both Figures 2 and 3 together, during AWVs, clinicians focus on what is in the best interests of patients, such as prevention of possible adverse events, by addressing their preventive service needs for both acute and chronic medical issues. In this study, the IHI’s 4Ms components and 6 key actions provided an overarching framework for starting to make sense of clinicians’ priorities in consideration of AWV components.

### Additional Analysis Related to Caring for Medicare Beneficiaries with MCI/ADRD

Table 5 summarizes the findings from Q3 to Q5. The themes of clinicians’ challenges regarding caring for patients with MCI/ADRD (Q3) were grouped into two overarching themes: (A) needing additional time to attend to patients’ issues and needed support, and (B) difficulties in assessing patients’ social and support needs and providing referrals.

**Table 5.** Clinicians’ Experiences Working with Patients with MCI/ADRD (n = 26; Open-Ended Questions #3-5).

Themes (abstracted from the open question)	Themes found in the transcripts (yes/no)	Frequency of each theme found in the transcripts (%)
*Please share with me your challenges when it comes to the health of your older adult patients with MCI/ADRD (Question #3; a total of 11 themes categorized into 2 overarching themes)*		
*Overarching Theme A. Needing additional time to attend to patients’ issues and needed support (TC#1)*		
A.1. Needing to check that the family is caring for the patient in a way that ensures that the patient is safe at home. Patients may need someone more actively engaged in ensuring all their aspects of life are OK and the interaction is positive (TC#1, TC#4, TC#5, TC#6)	Yes	9 (35)
	No	17 (65)
A.2. Patient’s poor adherence to the treatment recommendations or the plan made in the clinic, particularly for patients with a more severe degree of forgetfulness or cognitive impairment (TC#1, TC#5)	Yes	8 (31)
	No	18 (69)
A.3. Needing to assess difficulties in accessing care in a timely manner due to lack of reliable transportation. Seeing patients in their homes could increase access to care (TC#1, TC#6)	Yes	4 (15)
	No	22 (85)
A.4. Not enough clinic visit time to address patients’ multiple conditions and syndromes (TC#1)	Yes	3 (12)
	No	23 (88)
A.5. Needing to assess whether the patient is isolated or left alone and no one cares about what is happening (TC#1, TC#6)	Yes	3 (12)
	No	23 (88)
A.6. Needing to assess the caregivers’ physical capacity to help with activities of daily living. Often, the spouse of the patient is elderly too, and is not able to help the patient with activities of daily living (eg, bathing and hygiene) (TC#1, TC#6)	Yes	2 (8)
	No	24 (92)
A.7. Not enough time to build trust with patients with severe dementia and know what matters to them to facilitate meaningful communication (TC#1)	Yes	2 (8)
	No	24 (92)
A.8. Needing additional time to navigate the primary and secondary designees of the patient. Family members may have different ideas about how to care for the patient best and who gets to help with medical care-related decision-making (TC#1)	Yes	2 (8)
	No	24 (92)
*Overarching Theme B. Difficulties in assessing patients’ social and support needs and providing access (TC#6)*		
B.1. Having limited social workers to help access social/community resources. There are limited social/community support and resources available in Texas (TC#6)	Yes	4 (15)
	No	22 (85)
B.2. Difficulty obtaining the complete medical history of patients with advanced dementia. Patients’ family caregivers may not necessarily know everything that the patients are experiencing (TC#5, TC#6)	Yes	3 (12)
	No	23 (88)
B.3. Challenging to assess patients’ social and support issues and their long-term care planning. Social and support issues and needs often take precedence over their medical problems (TC#6)		
	Yes	2 (8)
	No	24 (92)
*Please share with me what you do at your practice location that allows you to recognize and diagnose people in the early stage of dementia (Question #4; 3 themes)*		
1. PCPs listen to patients’ and family members’ concerns about memory problems and assess for cognitive and daily function problems (yearly and as needed).	Yes	5 (19)
	No	21 (81)
2. If cognitive issues are found, clinicians proceed with further evaluation (referral to geriatrics or neurology) and address dementia issues with the patients and their family caregivers.	Yes	5 (19)
	No	21 (81)
3. Clinicians engage the patient and family caregivers in the new dementia diagnosis process. Clinicians go into detail. Clinicians talk to the patient.	Yes	4 (15)
	No	22 (85)
*Please share with me your opinion on how dementia diagnosis could be streamlined and caught sooner in older adults (Question #5; 5 themes)*		
1. Recognize when family caregivers report very early signs of something wrong (eg, cognitive or functional issues, changes in social functions).	Yes	9 (35)
	No	17 (65)
2. Follow-up family caregiver concerns with direct questions about the patient’s memory and the need for more formal cognitive screening.	Yes	9 (35)
	No	17 (65)
3. Do more AWVs and repeat memory tests to identify problems early, especially for patients 70 years and older.	Yes	7 (27)
	No	19 (73)
4. Set up timely follow-up or referral to a neurologist or dementia specialist to rule out/confirm memory or dementia diagnosis.	Yes	3 (12)
	No	23 (88)
5. Train clinic staff in the waiting area to capture issues/concerns with patients’ mental status or memory issues.	Yes	2 (8)
	No	24 (92)

Abbreviations: ADRD, Alzheimer’s Disease and Related Dementias; AWV, annual wellness visit; MCI, mild cognitive impairment; PCP, primary care provider; TC, thematic category.

Across Tables 3 to 5 (questions #1a, 1b, 3, 7-9), we identified 7 thematic categories (abbreviated as TC), as the overall thematic structure, which are: (TC#1) providing additional time to attend to patients’ issues and needed support; (TC#2) engaging primary care providers (PCPs) and trained non-PCP providers in expanding the AWV delivery capacity; (TC#3) scheduling and increasing patient/caregiver uptake needing PCPs’ in-person explanations of AWVs and encouragement during maintenance visits; (TC#4) assisting patients/caregivers in completing the AWV assessment questionnaire before AWVs to allow time for AWV providers to identify needs and develop/update the personalized prevention plan within the allotted time for each AWV; (TC#5) experiencing challenges in assessing patients’ risk factors and developing/updating the personalized prevention plan without a trusted caretaker to accompany patients with MCI/ADRD; (TC#6) assessing patients’ social and support needs and providing timely access; and (TC#7) affiliating with an existing referral network or health system providing timely access to clinical or non-clinical referrals or services. We included the corresponding TC number (TC#) after each theme.

In addition, the identified themes related to what clinicians do at the practice location that allows clinicians to recognize and diagnose people in the early stage of dementia (Q4), and their opinions on how a dementia diagnosis could be streamlined and detected sooner in older adults (Q5) are presented in Table 5.

We also asked clinicians their opinion of the new FDA-approved dementia care medications, Leqembi and Kisunla (Q10). Fourteen clinicians (n = 14, 53.8%) reported having no prior experience with the new medication, and 11 (n = 11, 42.3%) expressed that they did not have sufficient familiarity with the new medication(s) to recommend anything or have an opinion on them. A few clinicians expressed concerns about the possibility of the potential side effect of micro-bleeding in the brain (n = 2, 7.7%), safety concerns related to multiple uses of magnetic resonance imaging (n = 2, 7.7%), denial of coverage by health insurance companies for the infusion protocol (n = 3, 11.5%), and the high cost and time-consuming administration of these medications as barriers for patients receiving these new dementia care medications (n = 4, 15.4%). Four clinicians (n = 4, 15.4%) emphasized the balance between pharmaceutical and non-pharmaceutical interventions that may improve memory, reduce depression and anxiety, or the importance of maintaining good hydration and having a well-balanced diet.

## Discussion

This study gathered the views on the Medicare AWV from providers on the process, efficiency, and outcome of providing AWVs for patients with MCI/ADRD using the context of IHI’s 4Ms framework of an age-friendly health system. We interviewed 26 clinicians who had billed for at least one AWV to understand what does and does not work in AWVs for Medicare beneficiaries with MCI/ADRD. Most participants agreed or strongly agreed that AWVs improve health outcomes (n = 23, 88.5%) and reduce health disparities (n = 20, 76.9%) for Medicare beneficiaries with MCI/ADRD (Q1; Table 3). Regarding the improvement of health outcomes, participants believed that Medicare beneficiaries benefited from AWVs, with the degree of benefit varying according to the severity of their dementia. In a previous study,^14^ some PCPs questioned the utility of AWVs for patients with MCI/ADRD, a viewpoint that could affect access to preventive services and health outcomes for these patients.

Surprisingly, 23% of participants thought AWVs could not reduce health disparities. AWVs could help identify health disparities, but navigating limited resources and obtaining access to these social and community resources was challenging (Table 3). As observed, all 20 participants who agreed that AWVs reduced health disparities also agreed that AWVs improved health outcomes. As for the other 6 participants (23.1%) who did not agree that AWVs reduced health disparities, 3 of these 6 participants agreed that AWVs improved health outcomes. This observation suggests that the question of the agreement level at which AWVs reduce health disparities is independent of the question of the agreement level at which AWVs improve health outcomes.

Interviewees felt “What does not work” (Q8) was a lack of family caregivers’ engagement during AWVs, the need for additional time to perform in-depth screening and address identified issues, and a shortage of clinic-based social workers available to address non-medical problems (Table 4). These themes also revealed clinicians’ frustrations in connecting with family caregivers to identify issues (often non-medical) and help patients with MCI/ADRD and their family caregivers obtain needed resources. Patients with MCI/ADRD and their family caregivers often rely on effective primary care services, such as AWVs, for support and connection with resources to facilitate care coordination for dementia care.^43^ While expecting clinicians and clinics to resolve identified health disparities is not realistic, they can help patients and family caregivers access local resources. During AWVs, clinicians can identify sources of social determinants of health-related disparities (eg, transportation, dietary choices, access to needed specialists such as dietitians) and address these by working with clinic social workers and to make appropriate referrals.

Clinicians desired improvement in AWVs for Medicare beneficiaries with MCI/ADRD (Q9) by clearly explaining to patients and family caregivers what to expect from AWVs, putting additional effort into requiring a trusted family caregiver to attend the AWV with patients with MCI/ADRD, refining the clinic protocol to provide needed assistance to help patients or their family caregivers complete AWV questionnaires, offering AWVs via telehealth technology, and increasing clinic AWV delivery infrastructure to have non-PCP providers deliver AWVs (Table 4). Clinicians’ challenges when caring for patients with MCI/ADRD in ambulatory care settings (Q3) (ie, needing more time to assess patients’ issues and needing social worker support to evaluate patients’ social and support needs and provide access) corresponded with the findings from Q8 to Q9. They offered possible strategies for streamlining AWVs and care for patients with MCI/ADRD (Table 5). Clinicians’ strategies for recognizing people in the early stage of dementia (Q4-Q5) also offered approaches to improve AWVs, especially for those with an early MCI/ADRD diagnosis. These include listening to patients’ and family caregivers’ concerns about memory problems and engaging them in the new dementia diagnosis process, making timely referrals, providing coordination for dementia care, and repeating memory tests (Table 5).

Reflecting on our goal to identify successful and unsuccessful elements of AWV delivery for Medicare beneficiaries with MCI/ADRD and the lessons learned from clinicians’ viewpoints (Tables 3-5, questions #1a, 1b, 3, 7-9), the overall thematic structure emerged with coherence, revealing 7 thematic categories: (1) providing additional time to attend to patients’ issues and needed support related to IHI’s four 4Ms components and six key actions recommended by IHI for ambulatory care settings at least annually or after a change in condition^26,27^; (2) engaging PCPs and trained non-PCP providers in expanding the AWV delivery capacity; (3) scheduling and increasing patient/caregiver uptake needing PCPs’ in-person explanations of AWVs and encouragement during maintenance visits; (4) providing assistance to patients/caregivers in completing the AWV assessment questionnaire before AWVs to allow time for AWV providers to identify needs and develop/update the personalized prevention plan within the allotted time for each AWV; (5) experiencing challenges in assessing patients’ risk factors and developing/updating the personalized prevention plan without a trusted caretaker to accompany patients with MCI/ADRD; (6) assessing patients’ social and support needs and providing timely access; and (7) affiliating with an existing referral network or health system providing timely access to clinical or non-clinical referrals or services. This overall thematic structure further crystallized the findings from a recent scoping review^44^ regarding the implementation of AWVs for older adults with cognitive impairment, which suggests that effective AWV delivery for older adults with MCI/ADRD requires organizational readiness, structured implementation, and engaged health care providers in primary care settings (eg, team-based workflows, on-site social workers to support access to identified social and support needs).^44^

## Limitations

As a pilot, we used convenience sampling to recruit Texas clinicians who billed for at least 1 AWV to participate in a one-time virtual interview. The initial sampling of 250 clinicians was proposed in the original design of a regional study, which yielded very low participation. We then shifted to recruiting clinician participants through internal email announcements, which evolved into a single-institution study in Texas. This approach resulted in a strong institutional and self-selection bias as a study limitation. As a single academic institution study, the results may not be generalizable beyond this setting. Generalizing the findings to other contexts, study participants, or other settings should be done with caution. Our planned national clinician survey study will recruit up to 400 clinicians, which will expand diversity of clinician perspectives and also allow us to move beyond determining “what works and what does not work” for older adults living with dementia who receive AWVs to critically examining underlying differences between subgroups, such as by medical specialty (eg, family medicine, geriatrician, primary care internalist, nurse practitioner, and physician assistant) and practice location. Similarly, a larger sample will enable us to map the “What Matters” themes with the 4Ms and analyze the tensions, gaps, and overlaps among the 4Ms components.

Another limitation is that each interview lasted only an average of about 15 to 20 minutes, which is considerably short for a qualitative interview that explored complex systemic and experiential themes. Our pilot intended to keep the interview brief to encourage clinicians to participate, as well as to identify broad themes related to AWVs and refine the questionnaire instrument for use in the national survey. We understand that conducting short qualitative interviews may limit the richness of the data, particularly when examining health policy-level barriers. In addition, since this was a pilot study using inductive thematic saturation to develop the codebook for the national study, we did not conduct a power analysis to determine the sample size; instead, we recruited participants until thematic saturation was reached.

## Future Research Directions

Future qualitative studies should assess the roles of physicians and advanced practice clinicians versus non-prescriber health care providers (eg, wellness nurses) in AWV delivery across metropolitan and nonmetropolitan areas. Although successful, our local partnerships may not be applicable nationally, but we are expanding and building on our research partnerships with the Alzheimer’s Association, a national organization dedicated to advancing dementia practice, policy, and scholarship. Future studies should investigate how reframing recruitment messaging and materials can increase clinician recruitment. Also, to accelerate the practical implications of this study’s findings, further research, which includes conducting focus groups with primary care clinicians, is needed to validate the mapping of the IHI framework shown in Figures 2 and 3. In addition, in the parent project, we conducted two pilots, one solicited the perspectives of clinicians (the data used for the present pilot study), and the other solicited the perspectives of family caregivers, to understand “what works and what doesn’t” in AWVs for patients with MCI/ADRD in preparation for a larger national study. One of the future analyses will be to triangulate the perspectives of clinicians with those of caregivers, providing a more comprehensive and triangulated view. In this present paper, we focused on clinicians’ viewpoints on AWVs for older adults with MCI/ADRD.

## Implications

As a policy implication of this pilot study’s findings, CMS may consider allowing clinicians to tailor AWV components for Medicare beneficiaries by MCI/ADRD stage. Tailored AWVs for people with dementia could potentially motivate clinicians to optimize the allocated time for each AWV, which may result in improved health outcomes and a reduction in disparities in access to necessary care or resources. Information from clinicians’ insights can also guide CMS when making policy changes to strengthen AWVs. Incorporating IHI’s 4Ms framework for primary care settings^27,35^ and implementing science frameworks (eg, the Consolidated Framework for Implementation Research^45,46^) early on when examining the impacts of AWVs could lead to actionable findings and accelerate policy-related practice changes at the national-level as well as within local clinics or health systems. The feasibility and reimbursement implications of allowing clinicians to tailor AWV components by MCI/ADRD stage require additional research. Such research could involve conducting a series of focus groups to synchronize insights (eg, AWV documentation requirements) across Medicare, health system sectors, clinicians (eg, primary care providers caring for patients with MCI/ADRD), and family caregivers who have provided care to people with MCI/ADRD in the community or in assisted living or long-term nursing home settings.

Workforce readiness is an essential component of such policy changes and should be included in insight synchronization activities, such as using surveys or focus groups to solicit inputs from clinicians and educators who provide training to healthcare providers. CMS should consider providing funding to support small-scale quality improvement intervention studies in primary care clinics—especially those located in healthcare professional shortage areas and in rural communities where AWV is under-used. Such intervention studies can examine the feasibility and return-on-investment of tailored AWVs for people with dementia from the perspectives of payors, clinicians, community-dwelling patients with MCI/ADRD and their family caregivers, and community stakeholders (eg, professionals with aging in place expertise).

## Conclusions

Clinicians agreed that AWVs help improve health outcomes (n = 23, 88.5%) and reduce health disparities (n = 20, 76.9%) for Medicare beneficiaries with MCI/ADRD. Our study is the first to solicit clinicians’ insights on what works and what does not work about AWVs for patients with MCI/ADRD. CMS may consider allowing clinicians to tailor AWV components for Medicare beneficiaries by MCI/ADRD stage. Tailored AWVs for those patients with MCI/ADRD could encourage clinicians to optimize the allocated time for each AWV to address the unique needs of those with MCI/ADRD and their family caregivers.

## Supplemental Material

sj-docx-1-his-10.1177_11786329261432894 – Supplemental material for A Qualitative Study of a Pilot of Clinician Perspectives on the Delivery of Medicare Annual Wellness Visits for Patients with Dementia in an Academic Health Science Center in Texas Supplemental material, sj-docx-1-his-10.1177_11786329261432894 for A Qualitative Study of a Pilot of Clinician Perspectives on the Delivery of Medicare Annual Wellness Visits for Patients with Dementia in an Academic Health Science Center in Texas by Huey-Ming Tzeng, Yong-Fang Kuo, Monique R. Pappadis, Elizabeth A. Hennessy, Maribel M. Marquez-Bhojani, Samuel V. David, Elise Passy and Mukaila A. Raji in Health Services Insights
