# Supplementary material for: A Qualitative Study of a Pilot of Clinician Perspectives on the Delivery of Medicare Annual Wellness Visits for Patients with Dementia in an Academic Health Science Center in Texas
Source: Health Serv Insights. 2026 Mar 31;19:11786329261432894. doi: 10.1177/11786329261432894 (PMC13039569; doi:10.1177/11786329261432894)
Supplement: sj-docx-1-his-10.1177_11786329261432894 – Supplemental material for A Qualitative Study of a Pilot of Clinician Perspectives on the Delivery of Medicare Annual Wellness Visits for Patients with Dementia in an Academic Health Science Center in Texas [file sj-docx-1-his-10.1177_11786329261432894.docx]

A Qualitative Study of a Pilot of Clinician Perspectives on the Delivery of Medicare Annual Wellness Visits for Patients with Dementia in an Academic Health Science Center in Texas

**Supplemental Materials**

**eFigure S1.** Study flyer and interview guide

_________________________________________________________________________

**_______________________________________________________________________________________**

**Interview Guide (script for clinician interview)**

Good day! Hello, my name is XXX, and I am a researcher at the University of Texas Medical Branch at Galveston.

Can I please speak with Dr. __________ (clinician’s last name)? I am calling today because you previously agreed to help us with a study to see what works and does not work in Medicare’s Annual Wellness Visits for older adults with Alzheimer’s Disease and Related Dementias (ADRD) or mild cognitive impairment. You previously reached out to our team by (email/phone/letter) and spoke with (study coordinator) and conveyed your willingness to participate in this study. The study team also mailed you the materials related to this study. I hope you find the materials helpful. (Yes/No)

**Is now (this still) a good time for this interview?** (If yes, proceed. If no, thank you, I understand your need to reschedule for a better date/time. When will work best for you?)

Thank you for agreeing to help us understand more about the care received by these patients.

This interview includes 10 questions regarding your opinion as a clinician about the Annual Wellness Visit services you provide to dementia patients. It should take about 30 minutes.

I will record our conversation so I can focus on talking to you. The information you provide is considered confidential and private. This interview recording will only be reviewed by the study team members. Only summary data without your personal information will be reported in the research report.

Again, thank you for helping us understand more about preventive medicine services for dementia patients. We could not complete this study without you!

**The purpose of this study is to identify what works and what does not work in delivery of the** **Annual Wellness Visit for people with dementia.**

**Please share with me your insights and opinions of the Annual Wellness Visit and other preventive medicine services you provide to ADRD patients.**

1.Based on your best estimate, do you agree that Annual Wellness Visits can:

1.a. Improve the health outcomes of your older adult patients with dementia? (answer 1-5, with 1 as strongly disagree and 5 as strongly agree; please tell us more)

_01_ Strongly Disagree _02_ Disagree _03_ Neither Agree/Disagree

_04_ Agree _05_ Strongly Agree

Please briefly tell me why. _________

1.b. Reduce the health disparities of your older adult patients 65 years and older and with dementia? (answer 1-5, with 1 as strongly disagree and 5 as strongly agree; please tell us more)

_01_ Strongly Disagree _02_ Disagree _03_ Neither Agree/Disagree

_04_ Agree _05_ Strongly Agree

Please briefly tell me why. _________

2.Please share with me your priorities when it comes to the health of your older adult patients with dementia.

3.Please share with me your challenges when it comes to the health of your older adult patients with dementia.

4.Please share with me what you do at your practice location that allows you to recognize and diagnose people in early stage of dementia.

5.Please share with me your opinion on how dementia diagnosis could be streamlined and caught sooner in older adults.

6.Please share with me the preventive medicine services you have provided to older adults living with dementia (e.g., cognitive screening, medication review, mobility assessment, patient education, vaccinations, referrals to therapists or specialists, treatments, and advance care planning).

7.Please share with me your opinion on **what works** in your practice or health care system for older adults living with dementia who receive Annual Wellness Visits.

8. Please share with me your opinion **what does not** work in your practice or health care system for older adults living with dementia who receive Annual Wellness Visits.

9. Please share with me your opinion on what should happen to improve Annual Wellness Visit delivery for older adults living with dementia.

10. Please share with me your opinion of the new FDA approved dementia care medications (Leqembi/Lecanemab or Kisunla [Donanemab-azbt]).

Thank you so much for taking the time to help us understand more about the value of Annual Wellness Visits to you and your patients. I have just a few more **demographic questions**.

1.Dr. _____, may I know your credentials/specialty (choose all that apply)?

_01_ Family Medicine Physician

_02_ General Internal Medicine

_03_ Geriatrician

_04_ Nurse Practitioner

_05_ Physician Assistant

_06_ Preventive Medicine

_07_ Primary Care Physician

_08_ Self-describe: ______________________

2.Dr. _____, may I know your total years of practice? ______ in years

3.Dr. _____, in your best estimate, in the past 12 months, how many of your patients were older adults aged >65 years with cognitive impairment (such as having mild cognitive impairment or at least one dementia diagnosis)?

_01_ 0 (none) _02_ 1-25 _03_ 26-50 _04_ 51-75 _05_ 76-100 _06_ More than 100

4.Dr. ____, may I know the Zip Code and State of your practice (if there are multiple practice areas, please list all)?

Zip Code(s) of Practice: ___________________ State(s) of Practice: ____________

5.Your sex: _00_ Female _01_ Male _02_ Unknown

6.Dr. _____, how would you identify yourself in terms of ethnicity?

_01_ Hispanic or Latino/a/x

_02_ Non-Hispanic or Non-Latino

_03_ Unknown (I prefer not to disclose)

7.How would you identify yourself in terms of race? (choose all that apply):

_01_ White

_02_ Black

_03_ Asian

_04_ American Indian/Alaska Native

_05_ Hawaiian

_06_ More than one race

_07_ Unknown

_08_ Self describe: ___________

8.Dr. _____, may I know your age in years: _____ years

Dr. ______ (last name of the clinician), thank you again for participating in this interview to learn your opinion on the preventive medicine services you provide to patients with dementia. Dr. ______ (last name of the clinician), may the study team contact you for future studies?

_00_ No _01_ Yes

If you have any additional thoughts or questions about this interview, please do not hesitate to contact us.

Have a wonderful day!

_________________________________________________________________________________________
